# Wind-assisted sprint migration in northern swifts

**DOI:** 10.1016/j.isci.2021.102474

**Published:** 2021-05-20

**Authors:** Susanne Åkesson, Giuseppe Bianco

**Affiliations:** 1 Department of Biology, Centre for Animal Movement Research, Lund University, Ecology Building, 223 62 Lund, Sweden

**Keywords:** Ecology, Biological sciences, Zoology, Animals, Ethology

## Abstract

Long-distance migration has evolved repeatedly in animals and covers substantial distances across the globe. The overall speed of migration in birds is determined by fueling rate at stopover, flight speed, power consumption during flight, and wind support. The highest speeds (500 km/day) have been predicted in small birds with a fly-and-forage strategy, such as swallows and swifts. Here, we use GLS tracking data for common swifts breeding in the northern part of the European range to study seasonal migration strategies and overall migration speeds. The data reveal estimated overall migration speeds substantially higher (average: 570 km/day; maximum: 832 km/day over 9 days) than predicted for swifts. In spring, swift routes provided 20% higher tailwind support than in autumn. Sustained migration speeds of this magnitude can only be achieved in small birds by a combined strategy including high fueling rate at stopover, fly-and-forage during migration, and selective use of tailwinds.

## Highlights

• Geolocators were used to estimate migration speeds in Lapland-breeding common swifts.
• Migration was straighter and faster in spring than in autumn.
• Overall spring migration speeds were higher than predicted by theory.
• Fueling at stopover, fly-and-forage, and wind assistance enabled maximum speeds.

## Graphical abstract

**Figure:**
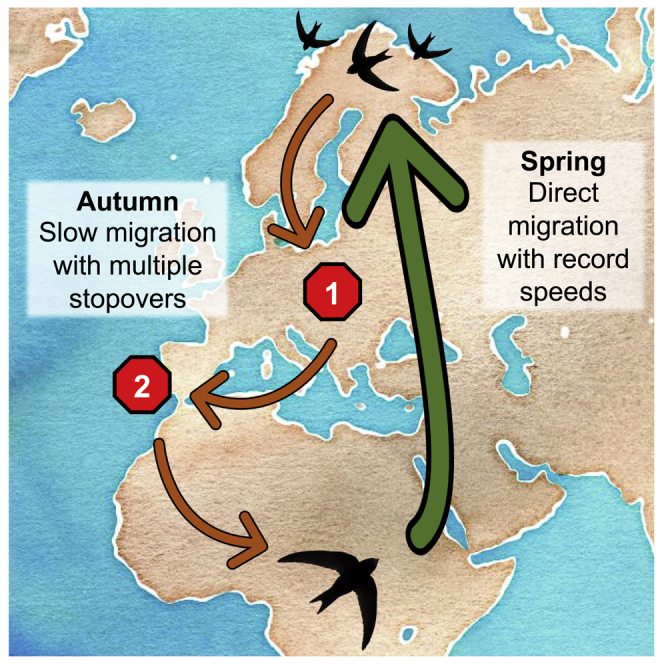

## Introduction

Birds have inhabited all continents on the planet and regularly perform some of the longest migrations recorded (Alerstam et al., 2003; Beason et al., 2012; Croxall et al., 2005; Gill et al., 2009; Stutchbury et al., 2009; Egevang et al., 2010; Bairlein et al., 2012; DeLuca et al., 2015; Sokolovskis et al., 2018). Tracking data show substantial variation in phenology, routes, and speed of migration, but for most birds, spring migration is faster than that in autumn (Nilsson et al., 2013). The overall migration speed is dependent on flight speed, fuel deposition rate and power consumption during flight (Hedenström & Alerstam 1997, 1998), and body size and flight style, with the highest speeds in smaller birds, typically 200–400 km/day (Alerstam et al., 2003; Hedenström and Alerstam 1998). For swallows and swifts that forage during flight on migration, the overall migration speed, including both fueling and flight, is predicted to be 500 km/day (Hedenström and Alerstam 1998). However, because of a maximum sustained metabolic scope (Hammond and Diamond 1997) and a high energy cost of flight (Pennycuick 1989), aerial insectivores may still need to rest for ca 25% of the time during migration (Hedenström and Alerstam 1998). Common swifts (*Apus apus*) spend up to 10 months on the wing during non-breeding (Hedenström et al., 2016), with a highly mobile lifestyle shared within the genus (Liechti et al., 2013; Hedenström et al., 2019), including regular high altitude flights (Hedenström et al., 2016, 2019; Dokter et al., 2013; Meier et al., 2018). Here, we use GLS tracking data for common swifts breeding in the northern part of their range to study seasonal migration strategies and overall migration speeds.

## Results and discussion

### Migration phenology

**Figure 1:**
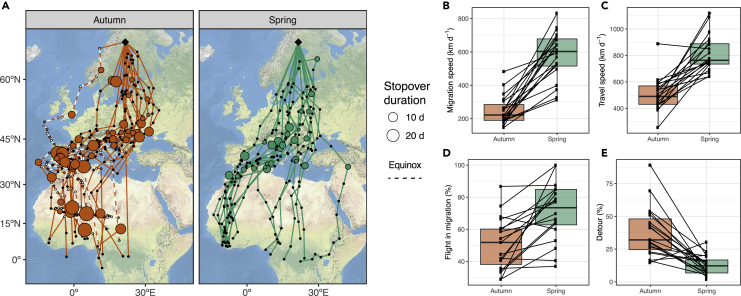
Routes and difference in migration performance for common swifts in autumn vs. spring (A) Routes from the breeding area in Swedish Lapland (black filled square) to sub-Saharan Africa depicted by miniature light-level geolocators (GLS). Lines connect 1-day GLS average locations (black dots), and open circles denote the location and duration of stopovers along the migratory routes. Locations affected by the equinox (unknown latitude) are shown by open dots and dashed lines. (B) Differences between autumn and spring migration in common swifts (n = 19). Boxplots show data distribution, and black lines connect the same individual during both migratory seasons. For averages and ranges, see Table 1; for individuals' performance, see Table S1. Statistics for autumn and spring migration, related to Figure 1.

**Figure 2:**
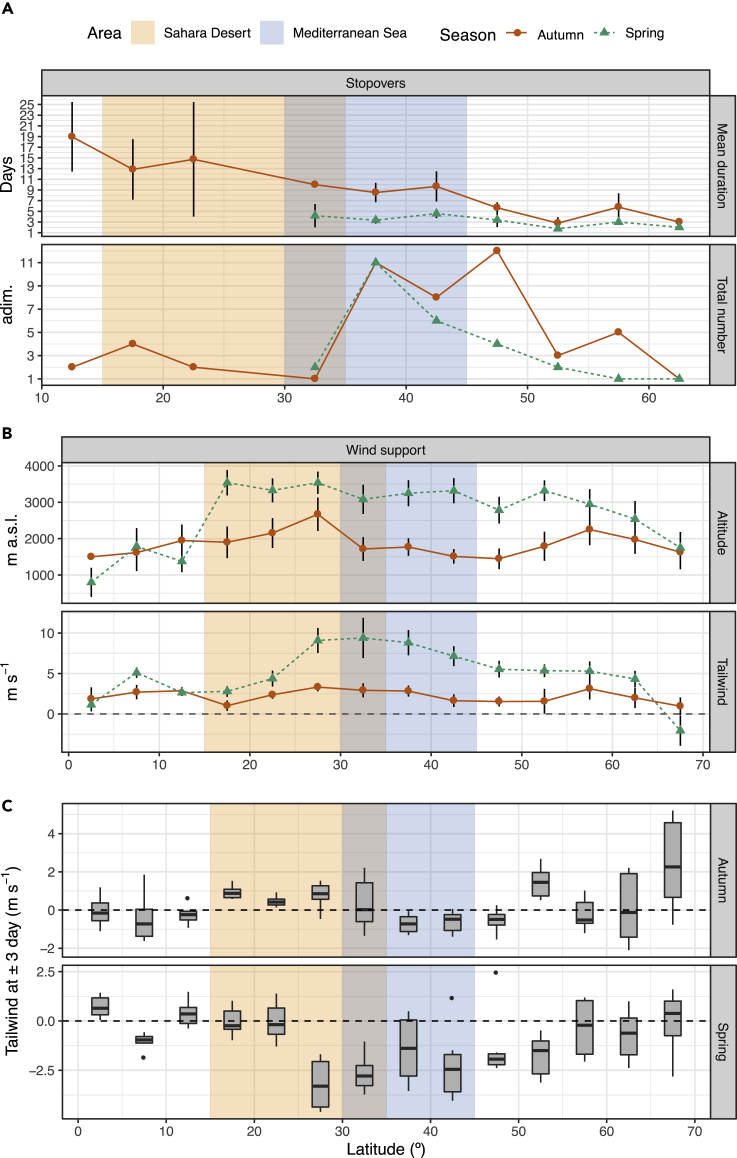
Migration performance and estimated wind support for common swifts as function of latitude (A) Mean duration (±SE) and total number of stopover periods. (B) Predicted flight altitude and tailwind support (±SE). (C) Predicted mean tailwind difference encountered by common swifts departing at ±3 days of their actual departure date. Negative values mean that the bird choosing a different departure day would have had on average less profitable winds at the given latitude. The approximate latitudinal extent of the ecological barrier including the Sahara Desert and the Mediterranean Sea is indicated by background shaded areas.

Common swifts remain airborne during non-breeding (Hedenström et al., 2016), enabling a highly mobile lifestyle and exploration of ephemeral food resources (Åkesson et al., 2012; Wellbrock et al., 2017; Boano et al., 2020). The timing of movements is important with respect to food availability and winds (Åkesson and Helm 2020; Wellbrock et al., 2017; Boano et al., 2020; Åkesson et al., 2016) and involves limited periods of fueling prior to autumn migration (Åkesson et al., 2012). Although these swifts spend most of their non-breeding year on the wing, their migrations are characterized by periods of relative geographic stasis (which we will call “stopovers”) interspersed with periods of concerted directional flight. Adult common swifts tracked by geolocation departed from the breeding sites in Swedish Lapland on average 15 August (standard deviation [SD]: ±10 days, range: 3 Aug-7 Sept, n = 19; Table 1). The swifts arrived at their wintering areas south of the Sahara approximately six weeks later by on average 26 September (SD: ±13 days, range: 31 Aug-14 Oct, n = 19; Table 1). Autumn migration involved 1–4 periods of residency (“stopover sites” with restricted geographic movements, hereafter “stopover”; Table 1) mainly located between 35° and 50° latitude, just before the crossing of the Sahara Desert (Figures 1A and 2A). Only 6 out of 19 birds engaged in a single stopover north of 55° latitude soon after departure in autumn. For 3 of these birds, the stop lasted only 2 days, and for the other 3 birds, the stops lasted 3, 8, and 15 days. Spring migration involving prolonged directed transportation flights started on average 14 May from the most northern site of residency south of the Sahara (SD: ±6 days, n = 19; Table 1) and lasted on average 15 ± 5 days. Swifts arrived to the breeding areas on average 29 May (SD: ±7 days, n = 19; range: 17 May-9 June, n = 20; Table 1) and remained resident at maximum 3 areas during spring migration, with 3 birds not performing any stopover at all (Table S1. Statistics for autumn and spring migration, related to Figure 1). Spring stopovers were predominantly located around the Mediterranean area (i.e., North Africa and Europe; Lat: 35°–45°) (Figures 1A and 2A).

**Table 1:** Migration characteristics for autumn (19 individuals) and spring (20) recorded by light-level geolocators for common swifts *Apus apus* breeding in Swedish Lapland

	Autumn			Spring		
	Mean	SD	Range	Mean	SD	Range
Departure date	15-Aug	10	3-Aug - 7-Sept	14-May	6	6-May - 29-May
Arrival date	26-Sep	13	31-Aug - 14-Oct	29-May	7	17-May - 9-June
Travel time (days)	20	7	10–46	10	2	7–15
Stopover time (days)	22	13	5–44	5	4	0–15
Total migration (days)	42	15	18–66	15	5	9–25
Total distance (km)	9933	1175	8000–12,025	7996	553	6594–9820
N stops	3	1	1–4	1	1	0–3
Detour (%)	38	19	15–89	13	7	2–30
Travel speed (km/day)	506	1129	256–888	816	138	641–1119
Migration speed (km/day)	250	88	147–483	570	143	312–832
Flight in migration (%)	51	16	29–87	71	18	37–100

The swifts spent significantly more days resident at stopovers during autumn migration (mean ± SD: 22 ± 13 days) than in spring (mean ± SD: 5 ± 5 days) (V = 190, n = 19, p < 0.001). Both total days spent on migration (t = 8.29, df = 18, p < 0.001) and the number of travel days, i.e., days in transportation flight (V = 190, n = 19, p < 0.001), were higher in autumn as compared to in spring (Table 1).

### Routes and migration strategy

The swifts left the breeding areas in Swedish Lapland toward southeast in autumn. Thereafter, they migrated south across northern Europe, where they shifted toward southwest to stopover sites on the Iberian Peninsula (Figure 1A). A lower number of swifts used stopover sites in south-eastern Europe, from Italy to Greece (Figure 1A). After stopover in the Mediterranean region, the swifts initiated broad front migration across the western and central parts of the Sahara Desert (Figure 1A; Åkesson et al., 2012). The crossing involved some staging time in the Sahel zone, before they reached the winter destinations in sub-Saharan West to Central Africa (Figure 1A).

Spring migration across the Sahara was initiated directly from the wintering sites or via a period of residency (stopover) spent in West Africa (Liberia; Åkesson et al., 2012) (Figure 1A). It took on average longer time (26 days) for the swifts to cross the Sahara in autumn from the Mediterranean region (i.e., approx. 40° latitude) to their final migration destination in the sub-Saharan region including stationary and directed migration flight segments (SD:±19 days, n = 19), than in spring from the departure location south of the Sahara to the Mediterranean Sea (7 days; SD: ±3 days, n = 19) (t = 4.34, df = 18, p < 0.001). There was also a difference in days spent in travel for the Sahara crossing between autumn (mean ± SD: 11 ± 8 days) and spring (mean ± SD: 5 ± 2 days) (t = 2.57, df = 18, p < 0.05).

The migration routes were significantly longer in autumn (mean ± SD: 9533 ± 1175 km) than in spring (mean ± SD: 7996 ± 553 km) (t = 5.09 df = 18, p < 0.001; Table 1), resulting in significantly longer detours calculated relative to a great circle route distance (Imboden and Imboden 1972) in autumn as compared to in spring (mean detour ± SD: 38 ± 19% in autumn, and 13 ± 7% in spring, t = 5.15, df = 18, p < 0.001; Figure 1E).

Our data reveal that common swifts breeding in the northernmost part of the European range migrate by more direct routes, i.e. shorter detours, in spring as compared to in autumn, but also following shorter detours in both seasons as compared to more southern populations (Åkesson et al., 2012, 2016). Still they cover substantial distances on migration (on average > 9900 km in autumn and >7900 km in spring), exceeding those recorded for populations in south and central Sweden (Åkesson et al., 2012). The initial part of the routes were mainly directed south across northern Europe in autumn as predicted by ringing recoveries (Fransson et al., 2008). The swifts used more and longer stopovers during autumn as compared to in spring, leading to a migration strategy combining prolonged flights and intermittent periods of residency, possibly including also daily foraging for fueling. An increase in stops occurred before the barrier crossing north of the Mediterranean region. The length of stopovers further increased with decreasing latitudes in autumn, with some of the longest noted in the northern Sahel zone on the southern border to the Sahara Desert, suggesting a transition to extended periods of residency during non-breeding allocated to this region (Åkesson et al., 2012). Likely, swifts were exploring good foraging conditions here before resumed migration (Åkesson et al., 2012, 2016; Norevik et al., 2019).

Only six birds explored autumn stopovers soon after departing from the breeding sites (>55° latitude, 2–15 days). This suggests initial fueling before departure from the breeding area (13 out of 19 birds), and for six swifts using initial stopover involving relatively short periods, and prolonged flights to stopover areas further to the south. The use of initial stopover may, however, also be affected by wind conditions met *en route* and not just the need to refuel. Our data suggest a short fueling period before migration is initiated, as compared to other long-distance avian migrants (Lindström 1991, 2003).

Stopover use during spring migration was very limited in our northern swifts, suggesting a migration strategy including foraging and flight along the way. This pattern suggests that the swifts minimize the overall time spent on spring migration (Alerstam and Lindström 1990). Three individuals did not use any stopover at all during spring migration, while the majority of the birds explored 1–3 staging areas, lasting on average 5 days, resulting in faster migration in spring than in autumn (Nilsson et al., 2013; Åkesson et al., 2012).

As support for a difference in migration strategy between seasons, we found more time spent in directed flight during spring (on average 71%) than in autumn (51%). The proportion of time spent on directed flight in spring approaches those limits predicted for swifts (ca 75%, Hammond and Diamond 1997) but will still enable swifts to include some periods for foraging and rest, the latter possibly by temporarily reaching higher altitudes (Hedenström et al., 2016, 2019). The activity limit is still relatively high and is restricted by maximum sustained metabolic scope (Hammond and Diamond 1997) and high energy cost of flight (Pennycuick 1989), leading to predicted need to rest for part (ca 25%) of the time during migration for aerial insectivores like swifts (Hedenström and Alerstam 1998). We find it interesting that northern swifts approach those predicted limits with respect to flight time and rest only on spring migration, but not in autumn, when they remain resident for longer periods and spend fewer days in directed flight. The stopovers prior to crossing of the Mediterranean Sea and the Sahara Desert in autumn (Figure 2) suggest fueling before resumed migration, which has also been noted for other populations of swifts (Åkesson et al., 2016).

### Migration and travel speeds

The swifts reached faster average overall migration speeds in spring (mean ± SD: 570 ± 143 km/day) as compared to in autumn (mean ± SD: 250 ± 88 km/day), including periods both at stopover and in directed transportation flight, but not including initial stopover (t = −9.24, df = 18, p < 0.001; Figure 1B). Travel speeds, excluding periods of stopover, were also faster in spring (mean ± SD: 816 ± 138 km/day) than in autumn (mean ± SD: 506 ± 129 km/day) (t = −7.17, df = 18, p < 0.001; Figure 1C). As a result, the swifts spent more time in directed transportation flight during spring migration (mean ± SD: 71 ± 18%) as compared to in autumn (mean ± SD: 51 ± 16%) (t = −4.67, df = 18, p < 0.001; Figure 1D).

A fly-and-forage strategy as suggested for swifts (Alerstam et al., 2003; Hedenström and Alerstam 1998) will lead to higher predicted migration speeds as compared to a strict migration and stopover strategy widely used by terrestrial birds, for which search and settling costs at stopover may be substantial and the cost of carrying large fuel reserves will be high (Alerstam et al., 2003; Hedenström and Alerstam 1998). However, the observed migration pattern suggests a mixed strategy, including prolonged stopover periods and daily foraging, but with different proportions for autumn and spring. The swifts kept higher migration and travel speeds in spring than in autumn, in line with what has been found in other avian migrants (Nilsson et al., 2013; Norevik et al., 2017; Meier et al., 2020). In fact, the overall migration speeds, not including initial fueling, during spring (570 km/day) exceed those predicted for swifts (500 km/day; Hedenström and Alerstam 1998). The high spring migration speeds further exceed those predicted for birds in general, taking size and maximum fueling rate into account (Alerstam et al., 2003). They are, furthermore, higher than those of other populations of common swifts (170 km/day in autumn and 336 km/day in spring Åkesson et al., 2012).

A challenge to correctly estimate migration speeds is the difficulty to estimate fueling rate and time spent fueling before migration is initiated (Lindström 1991; Lindström et al., 2019). Both factors have strong effects on calculations of overall migration speeds leading to erroneous estimations if not included (Lindström et al., 2019). To define time spent fueling and fueling rate is especially challenging for swifts, being airborne during non-breeding (Hedenström et al., 2016). If we use the estimation of initial period of residency in autumn (median: 2.5 days) recorded for the six swifts departing from the breeding area and making an initial stopover to predict fueling period, we may expect a high capacity for fueling since the time is short and only marginally slower estimations of migration speeds (a reduction by 6% in autumn and 15% in spring, with corresponding average migration speeds of 232 km/day and 485 km/day, respectively). Still, this time may be an underestimation of fueling period prior to spring migration, but since 17 out of 20 individuals initiated their migration from the wintering sites, we cannot estimate fueling time for spring migration from stopover time. Based on the above reasoning, we may double the number of days spent fueling prior to departure to 5 days, leading to a reduction of calculated overall migration speed by 27% in spring resulting in an average speed of 418 km/day. Since swifts may forage daily on their way and they have been shown not to substantially increase body mass prior to departure (Åkesson et al., 2012), we believe the estimations of migration speeds based directly on our data, including the estimated reductions generated by assuming periods of initial fueling (6% reduction in autumn and 15–27% reduction in spring), are realistic. At the same time, the migration speed remains high in relation to other bird migrants (Alerstam et al., 2003; Lindström 1991), but with the size and lifestyle of swifts, this can be expected (Alerstam et al., 2003; Hedenström and Alerstam 1998).

### Movements in relation to winds

Winds have strong impact on birds in air and especially during migrations when energy and time are minimized (Alerstam and Lindström 1990). Winds can add substantially positively or negatively to realized flight ranges (Liechti 2006). The estimation of strongest wind profit during migration was for both autumn and spring migratory seasons predicted between 25° and 45° of latitudes while crossing the northern part of the Sahara Desert and the Mediterranean Sea (Figure 2B). However, tailwind support was lower during autumn (2.6 ± 2.2 m s-1) as compared to spring migration (8.6 ± 5.5 m s-1) (t = −4.3, df = 18, p < 0.001). The maximum tailwind speed of 36 m s-1 (i.e., more than 3 times the assumed airspeed) was reached in spring around 30°–35° latitude at a flying altitude of 5,500 m a.s.l. Furthermore, autumn migration across the Sahara and the Mediterranean Sea was predicted at a significantly lower altitude than the spring migration (t = −5.5, df = 18, p < 0.001) with the average altitude in autumn of 1,883 ± 621 m a.s.l. compared to 3,421 ± 882 m a.s.l. in spring (Figure 2B).

We did not find any effect of departure day on the tailwind component that the bird would have encountered at their departure location in a ±3 days range (likelihood ratio test: χ^2^(5) = 5.76, p = 0.33) nor did we find any effect of interaction between departure day and migratory season (χ^2^(5) = 1.37, p = 0.93). Hence, the swifts could have departed any day during the considered week range (both in autumn and spring) without experiencing a significantly different wind condition at their departure location. However, when we considered the effect of departure decision on the tailwind component across the entire migratory route, we found significant support for the fixed effect of departure day but only when considering also the interaction between day and latitude (χ^2^(15) = 34.27, p < 0.01). This means that the departure decision was affecting the tailwind encountered en route, but with a magnitude, that is a function of latitude. Furthermore, the contribution of the fixed effects to the complete model revealed that interactions between departure date and latitude were only supported when including the interaction with the spring season (multiple interactions at p < 0.05). Hence, in autumn, departure decision did not have any effect on wind conditions en route but only in spring. Furthermore, the estimates of the model for the interaction with spring, departure, and latitude were mostly negative, meaning that the birds were choosing the departure day that resulted in the best wind support in the considered 1-week period.

To further detail in which latitudinal range swifts were more affected by their departure decision, we considered the average tailwind difference between any assumed departure date in the ±3 day range and the actual departure date. This analysis showed that swifts were particularly aided (i.e. negative tailwind differences) by their departure decision during spring between 25° and 55° latitudes while crossing the northern part of Sahara Desert and reaching central Europe (Figure 2C). Thanks to their departure decision in this latitudinal range, swifts were able to gain almost 20% of wind support as compared to their south passage in autumn across the same latitudinal range. This proportion approaches differences in migration speeds between spring and autumn reported for a range of bird species (Nilsson et al., 2013), suggesting that at least part of the difference in migration speeds between seasons could be related to selective use of tailwinds by birds migrating along similar routes across the Sahara as our swifts.

## Conclusions

Common swifts stay airborne during non-breeding (Hedenström et al., 2016). In short, they live their life in the air where they are continuously exposed to changing weather and winds, sometimes leading to so-called “weather migrations” where swifts occasionally leave the breeding areas on mass migrations in response to bad weather (Koskimies 1950; Hedenström and Åkesson 2017). In addition, wind speeds often exceed air speeds generated by powered flight in birds (Hedenström and Åkesson 2017; Pennycuick et al., 2013; Henningsson et al., 2009) and as such winds need to be continuously handled by swifts on the wing. On migration, winds may have a strong effect on bird migration including timing of migration, flight altitude, drift, and migration speeds (Liechti 2006; Alerstam 1979). Birds have been shown to explore tailwinds for timely departures with wind support during migrations (e.g. Richardson 1978; 1990; Åkesson and Hedenström 2000), suggesting a capacity to optimize their migration with respect to wind profit. Here, we show how much wind support could be gained by exploring winds at different altitudes, resulting in a 20% gain in spring as compared to in autumn.

### Limitations of the study

This study used geolocation by light (Global Location Sensing) to track the migration of common swifts, which result in limited precision for locations, especially in terrestrial environments due to shading (Lisovski et al., 2012, 2020; cf. Åkesson et al., 2016), and lack of latitude information during equinox periods. Future studies would benefit from using miniature Global Positioning System technology to track the movements of individual swifts and to include other populations of swifts with varying migration distances. Since wind (Figure 2C) could only explain part of the difference in overall migration speeds between seasons (about double the speed in spring as compared to in autumn), there may still be other factors that we have not been able to identify. One important factor could be availability of food, affecting fueling rates with strong implications for realized migration speeds (Lindström 1991, 2003), and which may vary between seasons and latitudes. Insect abundance across the season and geographical range would be interesting to include in the study, as well as the choice and quality of the food by foraging swifts. Although we have been able to reveal exceptionally high overall migration speeds in swifts breeding in the northern part of the European range and use of a mixed migration strategy including both fly-and-forage and stopover use, future tracking studies would benefit from recording actual flight altitudes and movements with higher resolution.

### Resource availability

#### Lead contact

Further information and requests for resources should be directed to and will be fulfilled by the lead contact, Susanne Åkesson (Susanne.Akesson@biol.lu.se).

#### Materials availability

This study did not generate new unique reagents.

#### Data and code availability

The data sets and code generated during this study are available at the tracking database at Lund University [www.canmove.lu.se].

## Methods

All methods can be found in the accompanying Transparent Methods supplemental file.

## Supplemental information

Document S1. Transparent methods, figure S1, and table S1
