# Supplementary material for: Wind-assisted sprint migration in northern swifts
Source: iScience. 2021 May 20;24(6):102474. doi: 10.1016/j.isci.2021.102474 (PMC8257983; doi:10.1016/j.isci.2021.102474)
Supplement: Document S1. Transparent methods, figure S1, and table S1 [file mmc1.pdf]

iScience, Volume *24*

Supplemental information

Wind-assisted sprint migration in northern swifts

Susanne Åkesson and Giuseppe Bianco

Supplemental Information

Transparent Methods

Experimental model and subject details

Permissions were given by the Malmö/Lund Ethical Committee for Scientific work on animals (Dnr 5.8.18-12719/2017). Permission to attach geolocators to common swifts in Sweden was given by Malmö-Lunds djurförsöksetiska nämnd (M112-09, M470-12). Permission to trap and ring common swifts in Sweden was given by the Swedish Nature Protection Agency and the Swedish Ringing Centre (nr 440) to SÅ.

Study site and capture of birds

In total 45 adult common swifts were captured by mistnets at the nest sites and equipped with miniature geolocators (GLS) in Hakkas (66.92°N, 21.55°E), Swedish Lapland in 2010, 2012 and 2014. In total 24 (53%) of the logged swifts were recaptured in later seasons, resulting in similar recapture rate as other colonies (Åkesson et al. 2012, 2016; Morganti et al. 2018). Out of these, 20 loggers recorded movements during one full year including both autumn and spring migrations for 20 unique individuals, but from these timing of autumn migration was missing out for one bird due to equinox problems (Table 1).

We used archival light loggers (Intigeo-W55B1 and W65B1) from Migrate Technology Ltd. without a stalk (Åkesson et al. 2012; Morganti et al. 2018). The logger was attached to the bird with a full body harness made of a soft braded nylon string, with one loop around the neck and each wing, respectively (Åkesson et al. 2012). The mass of the geolocator including harness (0.8-1.0 g depending on model), never reached above 3% of the body mass (Åkesson et al. 2012). We noted no signs on the skin or plumage indicating damage due to the attachment of the harness or geolocator at recapture.

Geolocation

For both geolocator models, we used the program *Intiproc* v.1.03 provided by the manufacturer Migrate Technology Ltd., to perform the initial linear correction function for the clock drift. A critical sun angle corresponding to a light-level value of 2 on the arbitrary geolocator light scale was used to minimize the difference in latitude between pre-and post-equinox, and at the same time minimize the uncertainty in latitude close to equinox for periods when the swifts were stationary as defined by the estimations of longitude. The "Hill-Ekstrom" procedure (Ekstrom 2004) was used to evaluate sun angle for each track as outlined in Åkesson et al. (2012). Depending on logger model, the sun angles varied between -3.0 to -4.0 and -5.0 to -6.8 degrees. From analyses of each trajectory, we excluded a five-week period around the autumn and spring equinoxes (i.e., two weeks prior to and three weeks after autumn equinox, and the reverse in spring), but used the longitude data to evaluate timing of movements as outlined in Åkesson et al. (2016). Due to the clean light

measurements (Åkesson et al. 2016), we could calculate a mean position for each day, and we used those positions for further analyses of route choice and timing of movement for the individual swifts.

Archival light-level geolocators attached to forest dwelling birds generate substantial errors in light measurements (latitude: 143±62 km, longitude: 50±34 km, mean±95% confidence interval; Fudickar et al. 2011; Lisovski et al. 2012), but since common swifts stay airborne during the non-breeding period (Hedenström et al. 2016), and the light sensor receives continuous light exposure, lower errors are expected as discussed in Åkesson et al. (2016).

Evaluation of movement data

Departure date from the breeding site was estimated based on a change in pattern of light data (i.e., extended dark periods at night, and regular visits in the nestbox during daytime when feeding young during breeding, to an abrupt change to continuous light in daytime and dark nights during migration). We evaluated the timing of departure from breeding and wintering sites, as well as stopover duration and speed of migration in autumn and spring. For each track we defined staging areas, i.e., stop-over sites where the swifts stayed on average >2 days with limited change in latitude and longitude positions, and movement segments where those positions changed between days, as outlined in Åkesson et al. (2016) and exemplified in Figure S1. Method to classify GLS data, Related to Figure 1. In autumn, movements sometimes coincided with the equinox period, and then we used only longitude to define periods of stopover and periods of movement, which was possible because of substantial longitude shifts during migration (Åkesson et al. 2016). For part of tracks around equinoxes, the trajectory was represented by using linear temporal interpolation of the latitudes as shown in Figure 1A.

We used the movement trajectories including positions for prolonged stopover sites, to calculate both overall migration speed (movements including stopover time; km/day) and travel speed (speed of movement during travel days; km/day) for autumn and spring migration. Locations of staging areas are given as the mean latitude and longitude positions for the time spent resident. The characteristics of movements during autumn and spring are given in Table 1.

Evaluation of wind profit and departure decisions

To evaluate the wind profit along the migratory routes, we first linearly interpolated the GLS positions at 6-hour interval to have constant timestamps that were also coinciding with the wind database temporal resolution (i.e., 00:00, 06:00, 12:00 and 18:00 UTC; see below). Then we annotated all locations with wind data from the NCEP/NCAR Reanalysis project (www.cdc.noaa.gov) using the RNCEP R package (Kemp et al. 2012a). Since GLS do not provide information on flight altitude, we used wind conditions at six pressure levels (i.e. 1,000, 925, 800, 700, 600 and 500 hPa), corresponding to approximately 100, 750, 1,500,

3,000, 4,200 and 5,500 m above sea level (a.s.l.). For each location and all pressure levels, we calculated the wind profit using the airspeed equation in the RNCEP package, assuming that swifts flew with fixed airspeed of 10 m s^{-1} (Hedenström & Åkesson 2017), and preferred direction coinciding with the direction to the next location along the track (Kemp et al. 2012b). Finally, for each location we selected the flight altitude corresponding to the pressure level that provided the strongest wind support expressed as the speed of the tailwind component. That is, the bird was allowed to change altitude every 6h to find the optimal performance at the given location. The predicted flight altitude and the corresponding tailwind speed were averaged at 5º latitude intervals along the tracks before plotting and statistical testing seasonal and regional differences as outlined below.

To infer departure decisions in relation to winds, we annotated all tracks' departures (from breeding area, wintering area or any stopover location) with wind data as outlined above. We calculated the potential wind profit as tailwind difference between any of the 3 days previous departure and 3 days after departure (i.e., one-week interval centred around the actual departure date) and the actual departure date. In this scenario, a negative tailwind difference means that in the considered day the bird would have received less support from the winds (i.e., the bird did the right decision not departing in this day) and a positive value would indicate that a better wind situation existed for departure than the one chosen by the bird. In this way, we could assess whether a departure decision was based on favourable wind conditions at the departure locations. Similarly, we calculated tailwind difference at ±3 days for each track at 5º latitude intervals. In this way we could also test whether departure decisions were rather based on favourable winds along the upcoming migratory leg and, hence, on the ability of the bird to predict wind conditions a few days ahead and in anticipated geographical locations several kilometres away from the departure location.

Quantification and statistical analysis

Analyses of migratory movement data were done with paired samples t-test or paired samples Wilcoxon test after a Shapiro-Wilk normality test. To assess the potential effect of departure decision on wind support during migration we built a series of linear mixed-effects models using the *lme4* package version 1.1-23 (Bates et al. 2015) and *lmerTest* 3.1-2 (Kuznetsova et al. 2017). To test whether departure decisions were made based on wind at the departure location we modelled the difference of tailwind support against the fixed effects of migratory season and departure day (±3 days range). We included in the model the interaction between season and day and bird ID as random factor to account for the repeated measures of the same individual. To assess the effect of departure decision along the migratory route we modelled the difference of tailwind support averaged in 5º of latitude bins including season, day, latitude and their interactions as fixed effects and bird ID as random effect. For both models, we evaluated the contributions of the fixed effects comparing the complete models above against a depleted model missing the specific fixed effect and/or its interactions with the likelihood ratio test (Bates et al. 2015). Maps, plots

and statistical analysis were performed in the software R ver. 3.6.3 (R Development Core Team 2020).

Supplemental references

Bates, D., Maechler, M., Bolker, B., & Walker, S. (2015). Fitting linear mixed-effects models using lme4. J. Stat. Software 67, 1e48.

Ekstrom, P.A. (2004). An advance in geolocation by light. Memoirs of the National Insitute of Polar Research, Special Issue, 58, 210–226.

Fudickar, A. M., Wikelski, M., & Partecke, J. (2011). Tracking migratory songbirds: accuracy of light-level loggers (geolocators) in forest habitats. Methods Ecol. Evol. 3, 47–52.

Kemp, M.U., Emiel van Loon, E., Shamoun-Baranes, J., & Bouten, W. (2012a). RNCEP: global weather and climate data at your fingertips. Methods Ecol. Evol. 3, 65–70.

Kemp, M.U., Shamoun-Baranes, J., van Loon, E.E., McLaren, J.D., Dokter, A.M. & Bouten, W. (2012b). Quantifying flow-assistance and implications for movement research. J. Theor. Biol. 308, 56-67.

Kuznetsova, A., Brockhoff, P.B. & Christensen, R.H.B. (2017). "lmerTest Package: Tests in Linear Mixed Effects Models." J. Stat. Software 82, 1–26.

Morganti, M., Rubolini, D., Åkesson, S., Bermejo, A., de la Puente, J., Lardelli, R., Liechti, F., Boano, G., Tomassetto, E., Ferri, M., Caffi, M., Saino, N., and Ambrosini, R. (2018). Effect of light-level geolocators on apparent survival of two highly aerial swift species. J. Avian Biol. 49, jav-01521.

R Development Core Team. (2020). R: a language and environment for statistical computing. Vienna (Austria): R Foundation for Statistical Computing. Available from: http://www.R-project.org.

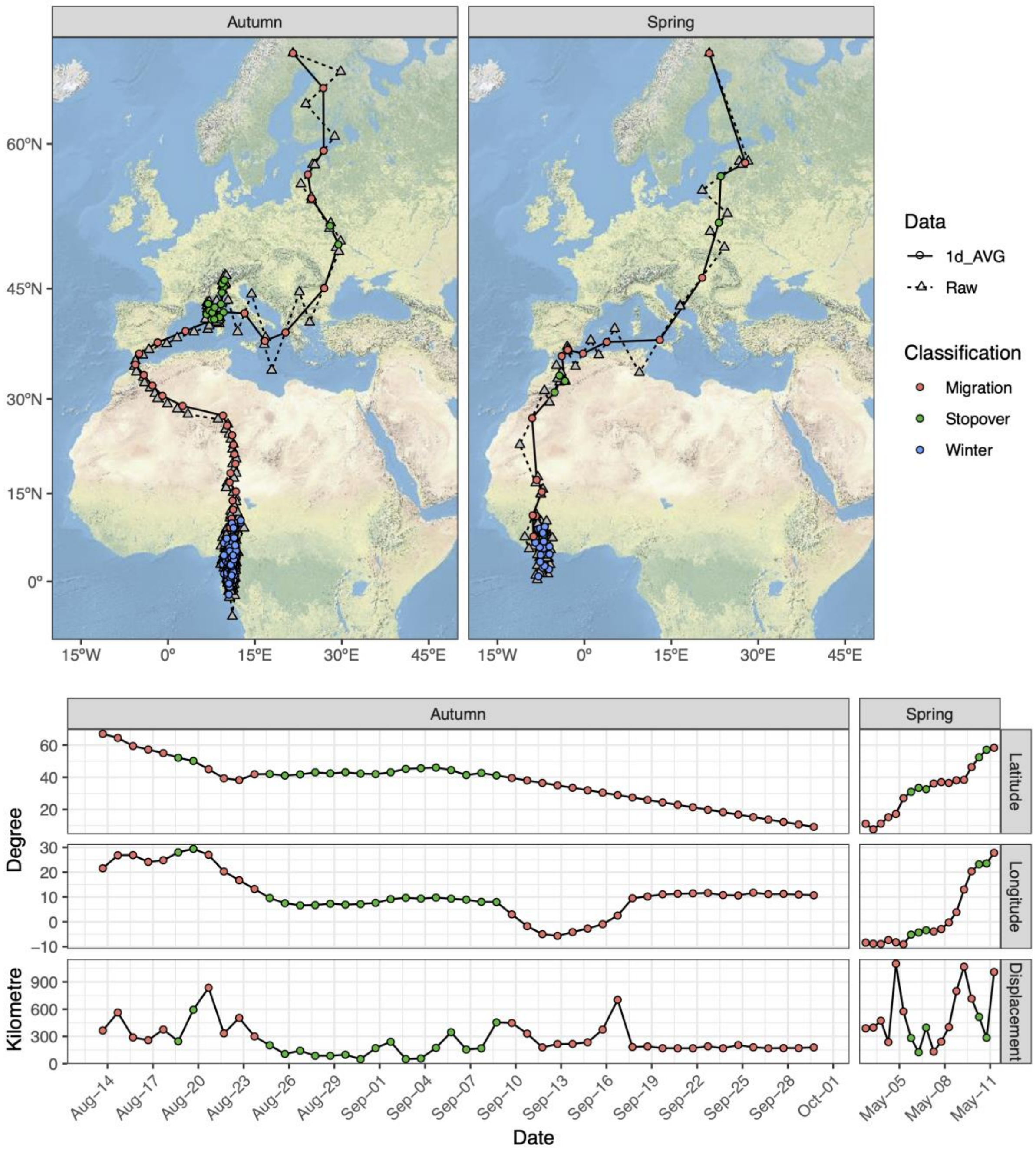

Figure S1. Method to classify GLS data. Related to Figure 1.

Example of the information used to classify movements from GLS data. Raw data (triangles) refers to locations obtained by light measurements twice a day. The two daily locations are averaged to obtain a daily location (circles) that define the migratory path. Stopovers are identified combining observation of locations (raw and 1d average) and daily changes in latitude, longitude and displacement. For illustration purpose latitude locations during equinoxes have been linearly interpolated.

Table S1. Statistics for autumn and spring migration. Related to Figure 1.
Data are for n=20 common swifts breeding in Swedish Lapland and wintering in sub–Saharan Africa.

	Season	ID	Departure	Arrival	Travel time	Stopover time	Total migration	Total distance	N stops	Detour	Travel speed	Migration speed	Flight in migration
1	Autumn	3213_A40431	04–Aug	10–Sep	20 d	17 d	37 d	8000 km	3	26 %	397 km/d	215 km/d	54 %
2	Autumn	3215_A32231	06–Aug	27–Sep	18 d	33 d	52 d	9922 km	4	53 %	537 km/d	191 km/d	36 %
3	Autumn	3219_A40439	16–Aug	07–Sep	16 d	5 d	21 d	8571 km	1	30 %	541 km/d	403 km/d	75 %
4	Autumn	3220_A32213	15–Aug	03–Oct	20 d	28 d	49 d	9197 km	2	41 %	451 km/d	188 km/d	42 %
5	Autumn	3224_A40442	12–Aug	14–Oct	18 d	44 d	62 d	9178 km	4	16 %	509 km/d	147 km/d	29 %
6	Autumn	3225_A32225	15–Aug	13–Oct	17 d	42 d	58 d	10362 km	2	43 %	616 km/d	177 km/d	29 %
7	Autumn	3226_A32224	21–Aug	14–Sep	14 d	10 d	24 d	8388 km	2	28 %	594 km/d	348 km/d	59 %
8	Autumn	3228_A40448	09–Aug	29–Sep	21 d	30 d	51 d	9101 km	3	32 %	425 km/d	178 km/d	42 %
9	Autumn	3229_A40451	06–Aug	16–Sep	18 d	22 d	40 d	10420 km	3	51 %	582 km/d	259 km/d	44 %
10	Autumn	3231_A40453	16–Aug	25–Sep	23 d	16 d	39 d	9859 km	2	30 %	420 km/d	251 km/d	60 %
11	Autumn	3232_A32245	07–Aug	12–Oct	22 d	44 d	66 d	10311 km	4	45 %	470 km/d	157 km/d	33 %
12	Autumn	A260_A31257	07–Sep	08–Oct	21 d	10 d	31 d	8057 km	3	23 %	388 km/d	258 km/d	66 %
13	Autumn	A269_A31256	13–Aug	31–Aug	10 d	8 d	18 d	8455 km	1	16 %	888 km/d	483 km/d	54 %
14	Autumn	A272_A31267	02–Sep	02–Oct	20 d	10 d	30 d	9332 km	3	42 %	466 km/d	316 km/d	68 %
15	Autumn	A283_A31260	29–Aug	29–Sep	19 d	13 d	32 d	8380 km	1	15 %	436 km/d	264 km/d	61 %
16	Autumn	A288_A31266	14–Aug	14–Oct	25 d	21 d	61 d	12025 km	4	89 %	490 km/d	199 km/d	41 %
17	Autumn	A295_A31265	07–Aug	29–Sep	46 d	7 d	53 d	11713 km	3	69 %	256 km/d	222 km/d	87 %
18	Autumn	A306_A31264	13–Aug	17–Sep	18 d	17 d	35 d	10803 km	2	54 %	591 km/d	306 km/d	52 %
19	Autumn	N531_A31261	03–Aug	20–Sep	16 d	31 d	47 d	9058 km	2	22 %	557 km/d	192 km/d	34 %
20	Spring	3213_A40431	19–May	04–Jun	11 d	5 d	15 d	7928 km	2	20 %	747 km/d	521 km/d	70 %
21	Spring	3215_A32231	15–May	07–Jun	9 d	15 d	24 d	7737 km	3	7 %	883 km/d	326 km/d	37 %
22	Spring	3219_A40439	09–May	20–May	12 d	0 d	12 d	7497 km	0	4 %	641 km/d	641 km/d	100 %
23	Spring	3220_A32213	13–May	06–Jun	10 d	15 d	25 d	7712 km	2	9 %	770 km/d	312 km/d	40 %
24	Spring	3221_A40440	12–May	21–May	9 d	0 d	9 d	6594 km	0	2 %	733 km/d	733 km/d	100 %
25	Spring	3224_A40442	10–May	31–May	10 d	11 d	21 d	7859 km	2	20 %	776 km/d	374 km/d	48 %
26	Spring	3225_A32225	14–May	24–May	10 d	0 d	10 d	7696 km	0	14 %	745 km/d	745 km/d	100 %
27	Spring	3226_A32224	23–May	05–Jun	9 d	4 d	13 d	7918 km	1	7 %	923 km/d	613 km/d	66 %
28	Spring	3228_A40448	06–May	20–May	12 d	3 d	15 d	8395 km	1	16 %	693 km/d	570 km/d	82 %
29	Spring	3229_A40451	10–May	30–May	10 d	9 d	20 d	7887 km	2	12 %	754 km/d	399 km/d	53 %
30	Spring	3231_A40453	12–May	21–May	8 d	1 d	9 d	7611 km	1	6 %	977 km/d	832 km/d	85 %
31	Spring	3232_A32245	06–May	17–May	7 d	4 d	11 d	7643 km	1	12 %	1092 km/d	696 km/d	64 %
32	Spring	A260_A31257	29–May	09–Jun	9 d	2 d	11 d	7859 km	1	16 %	905 km/d	702 km/d	78 %
33	Spring	A269_A31256	22–May	04–Jun	9 d	4 d	13 d	7837 km	1	5 %	849 km/d	593 km/d	70 %
34	Spring	A272_A31267	20–May	31–May	7 d	4 d	11 d	7552 km	2	7 %	1119 km/d	672 km/d	60 %
35	Spring	A283_A31260	16–May	05–Jun	15 d	5 d	20 d	9820 km	2	30 %	644 km/d	497 km/d	77 %
36	Spring	A288_A31266	07–May	20–May	11 d	2 d	13 d	8445 km	1	19 %	757 km/d	642 km/d	85 %
37	Spring	A295_A31265	10–May	22–May	10 d	1 d	12 d	7675 km	1	7 %	736 km/d	643 km/d	87 %
38	Spring	A306_A31264	15–May	31–May	13 d	4 d	17 d	8810 km	2	23 %	671 km/d	529 km/d	79 %
39	Spring	N531_A31261	17–May	01–Jun	10 d	5 d	15 d	8039 km	2	15 %	820 km/d	531 km/d	65 %
